# Emerin Phosphorylation during the Early Phase of the Oxidative Stress Response Influences Emerin–BAF Interaction and BAF Nuclear Localization

**DOI:** 10.3390/cells9061415

**Published:** 2020-06-06

**Authors:** Vittoria Cenni, Stefano Squarzoni, Manuela Loi, Elisabetta Mattioli, Giovanna Lattanzi, Cristina Capanni

**Affiliations:** 1 CNR Institute of Molecular Genetics “Luigi Luca Cavalli-Sforza”, Unit of Bologna, 40136 Bologna, Italy; vittoria.cenni@cnr.it (V.C.); squarzoni@area.bo.cnr.it (S.S.); manuk986@gmail.com (M.L.); emattiol@area.bo.cnr.it (E.M.); lattanzi@area.bo.cnr.it (G.L.); 2 IRCCS Istituto Ortopedico Rizzoli, 40136 Bologna, Italy; 3 Department of Biomedical and Neuromotor Sciences, University of Bologna, 40127 Bologna, Italy

**Keywords:** emerin, EDMD1, BAF, BANF1, laminA/C, laminopathies, chromatin, prelamin A, DNA-damage response, oxidative stress

## Abstract

Reactive Oxygen Species (ROS) are reactive molecules required for the maintenance of physiological functions. Oxidative stress arises when ROS production exceeds the cellular ability to eliminate such molecules. In this study, we showed that oxidative stress induces post-translational modification of the inner nuclear membrane protein emerin. In particular, emerin is phosphorylated at the early stages of the oxidative stress response, while protein phosphorylation is abolished upon recovery from stress. A finely tuned balance between emerin phosphorylation and *O*-GlcNAcylation seems to govern this dynamic and modulates emerin–BAF interaction and BAF nucleoplasmic localization during the oxidative stress response. Interestingly, emerin post-translational modifications, similar to those observed during the stress response, are detected in cells bearing *LMNA* gene mutations and are characterized by a free radical generating environment. On the other hand, under oxidative stress conditions, a delay in DNA damage repair and cell cycle progression is found in cells from Emery–Dreifuss Muscular Dystrophy type 1, which do not express emerin. These results suggest a role of the emerin–BAF protein platform in the DNA damage response aimed at counteracting the detrimental effects of elevated levels of ROS.

## 1. Introduction

Oxidative stress is defined as an imbalance between the production of reactive free radicals and the efficacy of antioxidant defenses. Production of free radicals is a physiological event taking place at the cellular level. These free radicals are important mediators of responses leading to cellular migration, differentiation, and proliferation; however, when cells become unable to control the amount of free radicals, oxidative stress arises [1]. In this regard, to avoid excessive Reactive Oxygen Species (ROS) accumulation, cells have developed several antioxidant systems, including enzymatic and non-enzymatic mechanisms, that limit protein, lipid, and DNA oxidation [2]. It is well known that an increase in free radical production and/or a decrease in the cellular defenses against ROS is a common event during cellular senescence [1]. Interestingly, a similar metabolic status occurs as a result of genetic mutations affecting some nuclear envelope proteins [3,4,5,6].

Laminopathies are a group of rare genetic disorders due to mutations in proteins mainly located at the nuclear membrane or the nuclear lamina [7]. Several different phenotypes characterize laminopathies: muscular dystrophy, cardiomyopathy, neuropathy, lipodystrophy, and accelerated aging (progeria).

Most laminopathies are due to mutations in the *LMNA* gene, encoding lamin A and lamin C as major splicing products. Lamin A/C are type V intermediate filaments that, in combination with lamin B, form a proteinaceous mesh underlying the inner nuclear membrane referred to as the nuclear lamina [8]. Differently from lamin C, lamin A is produced from a protein precursor, prelamin A. This 74-kD protein undergoes post-translational modifications comprising of C-terminal farnesylation, carboxymethylation, and proteolytic cleavage, which determine the removal of the prelamin A-specific C-terminus sequence and the release of mature lamin A [8]. Some *LMNA* gene mutations, or mutations affecting the prelamin A endoprotease ZMPSTE24, impair prelamin A processing with consequent accumulation of diverse immature protein forms [9]. In particular, in Hutchinson–Gilford Progeria Syndrome (HGPS), a truncated prelamin A form, named progerin, is accumulated as a result of a mutation affecting a residue recognized by ZMPSTE24 [10,11]. On the contrary, in Restrictive Dermopathy (RD) and Mandibuloacral Dysplasia type B (MADB), prelamin A accumulation arises from mutations of the ZMPSTE24 metalloproteinase [12,13], while, in Familial Partial Lipodystrophy (FPLD) and Mandibuloacral Dysplasia type A (MADA), the underlying cause of prelamin A accumulation is unknown [7,14]. It has been previously observed that FPLD, HGPS, and RD cells are characterized by a “ROS-generating environment” [3,4], a peculiar metabolic status also detected in lamin A/C depleted cells [15,16]. Interestingly, the study of the nuclear envelope composition of laminopathic cells harboring a nonsense *LMNA* gene mutation demonstrated that the absence of A-type lamins affects not only nuclear lamina organization but also some characteristics of major lamin-binding proteins. In particular, in *LMNA* null cells, phosphorylation of emerin was increased [16].

Emerin is an inner nuclear membrane protein, mutated in type 1 Emery–Dreifuss Muscular Dystrophy (EDMD1) [17]. Emerin interacts with nuclear membrane and nuclear lamina proteins. In this regard, emerin interaction with lamin A/C, prelamin A and progerin (a mutated form of prelamin A) has been well documented [18,19,20]. Barrier-to-Autointegration Factor (BAF) is one of the best characterized emerin binding partners. It is a 21-kD protein located both in the cytoplasm and the nucleus where it can potentially recruit chromatin regulators and DNA damage response molecules [21]. The emerin–BAF interaction is governed by the presence of a LEM protein domain located at the N-terminal region of emerin. This protein sequence binds efficiently to BAF, even if emerin or BAF modifications can further influence the stability of the emerin–BAF complex [22,23]. In general, emerin phosphorylation decreases its binding to BAF while *O*-GlcNAcylation seems to have the opposite effect [24].

In this study, we showed that, upon induction of oxidative stress, emerin phosphorylation increases and influences the emerin–BAF interaction and BAF nuclear localization. Our data suggest a role of the emerin–BAF protein platform in the DNA damage response and ROS protection.

## 2. Materials and Methods

### 2.1. Cell Cultures, Transfection and Treatments

HeLa cells, HEK293 cells, and human skin fibroblasts were cultured at 37 °C with 5% CO_2_ in Dulbecco’s Modified Eagle’s Medium (DMEM) containing 10% heat inactivated fetal calf serum (FCS), 2 mM l-glutamine, 50 µg/mL penicillin, and 50 µg/mL streptomycin [25,26]. Control, EDMD1, and HGPS fibroblasts were obtained from the BioLaM biobank. The experimental protocol had been approved by the local ethical committee (Rizzoli Orthopedic Institute Ethical Committee approval Prot. Gen. 0018250—2016) and followed EU rules. 

**Figure 1:**
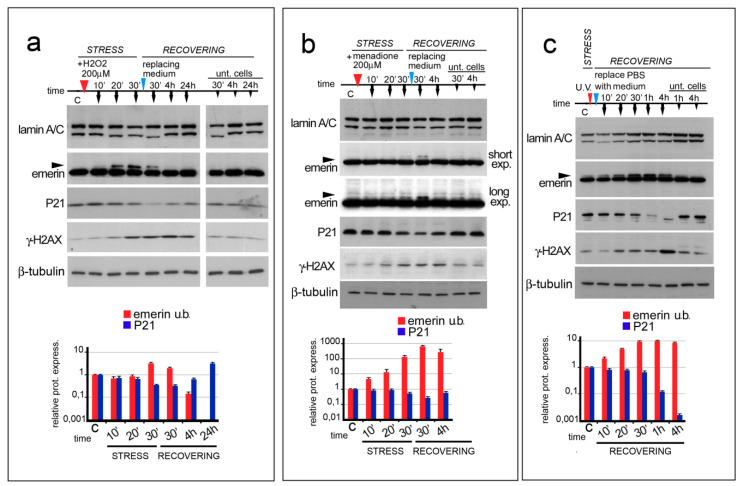
ROS generating agents affect emerin migration in SDS-PAGE. In (**a**–**c**), the time course and sample treatment is indicated over the Western blot image. Stress (STRESS) indicates the time (30 min) during which cells underwent stressing stimulus. Red arrowheads indicate H_2_O_2_ or menadione addition to the growth medium or cellular treatment with UV irradiation. Recovering (RECOVERING) indicates the time after replacement of growth medium (blue arrowheads) to remove H_2_O_2_ or menadione or PBSX1 replacement with growth medium, in the case of UV irradiation. Sample collection at different time-points is indicated by black arrows. Time (time) is indicated in minutes during the stress (10′, 20′, and 30′) and in minutes (30′) and hours (4 and 24 h) during the recovery. “C” indicates untreated cells (T^0^). “Unt. Cells” indicates untreated cells subjected only to growth medium replacement and harvested after 30 min, 4 h, and 24 h of recovering (30′, 4 h, and 24 h). (**a**) Western blotting analysis of lamin A/C, emerin, P21, gamma-H2AX, and beta-tubulin performed on protein lysates isolated from HeLa cells treated with 200 μM H_2_O_2_. Arrowhead indicates emerin upper band. (**b**) Western blotting analysis of lamin A/C, emerin, P21, gamma-H2AX (γ-H2AX), and β tubulin performed on protein lysates isolated from HeLa cells treated with 200 μM menadione. Two different exposures (short exp. and long exp.) of emerin immunolabeled bands are shown. Emerin upper band is indicated by arrowheads. (**c**) Western blotting analysis of lamin A/C, emerin, P21, gamma-H2AX (γ-H2AX), and β-tubulin performed on protein lysates isolated from HeLa cells treated with UV irradiation. Arrowhead indicates emerin upper band. In (**a**–**c**), the densitometric analysis of immunolabeled bands (logarithmic scale) is reported at the bottom of each panel. Red: upper emerin band; light blue: p21; blue: gamma-H2AX. Data are the means of three independent experiments.

**Figure 2:**
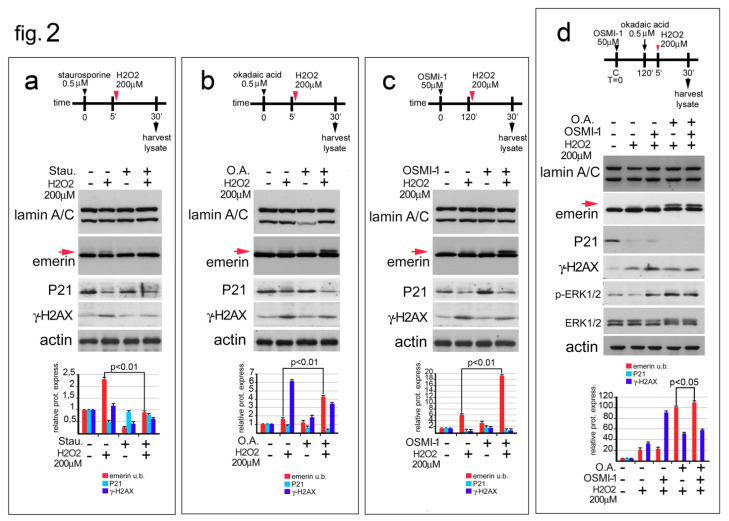
Emerin phosphorylation increase during the oxidative stress response. (**a**) Staurosporine affects the molecular weight of emerin molecular weight during oxidative stress. The experimental workflow showing the initial treatments (black arrowhead for staurosporine, red arrowhead for H_2_O_2_), and sample harvesting (black arrow) are indicated. Western blotting performed on protein lysates isolated from untreated HeLa cells (-), HeLa cells treated with H_2_O_2_ (H_2_O_2_) (+), and HeLa cells treated with staurosporine (Stau.), alone or in combination with H_2_O_2_, are shown. Lamin A/C, emerin, P21, gamma-H2AX (γ-H2AX) and actin immunological bands are shown. The upper (phosphorylated) emerin band is indicated (emerin u.b., red arrow). (**b**) Okadaic Acid favors the accumulation of the upper emerin band during the oxidative stress response. The experimental workflow showing start of treatment, (black arrowhead for Okadaic Acid, red arrowhead for H_2_O_2_), and sample collection (black harrow) are indicated. Western blotting was performed on protein lysates isolated from untreated HeLa or HeLa cells treated with H_2_O_2_ (H_2_O_2_) and Okadaic Acid (O.A.), either alone or in combination as shown. Lamin A/C, emerin, p21, gamma-H2AX (γ-H2AX), and actin immunoblotted bands are shown. Emerin phosphorylated (upper) band is indicated (emerinu.b, red arrow). (**c**) OSMI-1, a specific inhibitor of the O-GlcNAc transferase, affects the molecular weight of emerin during the oxidative stress response. The top of the panel shows the experimental workflow. Treatment start (black arrowhead for OSMI-1, red arrowhead for H_2_O_2_) and samples collection (black arrow) are indicated. Western blots of protein lysates isolated from untreated HeLa cells or HeLa cells treated with H_2_O_2_ (H_2_O_2_) or with OSMI-1 (OSMI-1), alone or in combination, are shown. Lamin A/C, emerin, p21, gamma-H2AX (γ-H2AX) and actin bands are shown. The red arrow indicates the upper (phosphorylated) emerin band. (**d**) Inhibition of both protein dephosphorylation and *O*-GlcNAcilation favors emerin phosphorylation during the oxidative stress response. The experimental procedure is reported. Treatment, (black arrowhead for OSMI-1, black arrow for O.A. and red arrowhead for H_2_O_2_) and sample collection are indicated. Western blotting analysis of protein lysates isolated from HeLa cells treated with H_2_O_2_ (H_2_O_2_), H_2_O_2_ plus OSMI-1, H_2_O_2_ plus O.A., or a combination of all treatments (H_2_O_2_ + O.A. + OSMI-1) is shown. Lamin A/C, emerin, P21, gamma-H2AX (γ-H2AX), and actin bands are shown. The red arrow indicates the phosphorylated emerin band. In (**a–d**), densitometric analysis of immunolabeled bands is reported at the bottom of the panel. Statistically significant difference (Student’s t-test), with respect to H_2_O_2_-treated cell values, is indicated. Red: emerin upper band; light blue: P21; blue: gamma-H2AX.

HEK293 cells were transiently transfected with full length FLAG-tagged prelamin A (LA-WT, pCI mammalian expression vector) and mutated constructs LA-C661M, LA-L647R, and LA-∆50 using FuGene reagent (Roche) [25]. HeLa cells were transiently transfected with His-tagged BAF using TransIT^®^-LT1 (Mirus) [27]. Transfected cells were evaluated (biochemical and immunofluorescence analyses) 48 h after transfection. The HeLa *LMNA* (LMNA−/−) and *ZMPSTE24* (ZMPSTE24−/−) knockout cell lines were generated using CRISPR-Cas9 mediated genome editing technology. The guide RNA sequence which targets the first exon of the gene was 5′- CCTTCGCATCACCGAGTCTGAAG-3′ for *LMNA* [28] and 5′-GGCCGAGAAGCGTATCTTCGGGG-3′ for *ZMSPTE24* as described before [29]. Constructs containing the Cas9 nuclease and selection markers were obtained from Addgene (#48138 and 48139) and published protocols were followed [30]. Control cells (*LMNA* +/+ and *ZMPSTE24*+/+) underwent the same treatment with a construct containing no guide RNA. The accumulation of prelamin A was obtained using 10 μM mevinolin (Sigma, Kawasaki, Japan) in growth medium for 18 h [25]. Treatment with ionizing agents were performed as follows. After 24 h of culture, cells were treated with H_2_O_2_ (200 μM) or Menadione (200 μM) for 30 min at 37 °C and then allowed to recover at 37 °C for 30 min up to 24 h by replacing treatment-containing medium with fresh medium [31,32]. Samples were collected at different time-points during H_2_O_2_ or Menadione treatment, as well as during the cell culture recovery period (see Figure 1a,b). For UV irradiation (Bio-Rad trans-UV, 302 nm), the growth medium was replaced with PBS1X and the cells were irradiated for 90 s. Afterwards, PBS1X was replaced with fresh medium and cells were harvested at the indicated times (see Figure 1c). Doxorubicin (2 μM) was added to the growth medium and cells were harvested as indicated in Figure S1b. To asses emerin secondary modification, cells were pretreated for 5 min with Staurosporine (0.5 μM) or Okadaic Acid (0.5 μM) in DMEM-10%FCS at 37 °C, H_2_O_2_ was then added to the growth medium and the cells were left under combined treatment for an additional 30 min. In a similar fashion, cells were pretreated with OSMI-1 (50 μM) [33] in DMEM-10%FCS at 37 °C for 2 h, then H_2_O_2_ was added to the growth medium. Cells were harvested as indicated in Figure 2.

### 2.2. Western Blotting and Immunoprecipitation

For Western blotting analysis cells were processed in lysis buffer containing 20 mM Tris-HCl, pH 7.5, 1% SDS, 1 mM Na3VO4, 1 mM PMSF, 5% β-mercaptoethanol and protease inhibitors. Samples were subjected to SDS gradient gel (5–20%) electrophoresis and transferred to nitrocellulose membrane overnight at 4 °C. Incubation with primary antibodies was performed for the indicated time. Bands were revealed using the Amersham ECL detection system and analyzed with ImageJ. For immunoprecipitation assays, transfected HeLa cells were lysed in buffer containing 50 mM Tris-HCl, pH 8.0, 150 mM NaCl, 1% NP40, 0.1% SDS, and protease inhibitors. Lysates were incubated with specific antibodies (0.5 μg anti-His-antibody) overnight at 4 °C. After the addition of 30 μL of protein A/G conjugated sepharose beads (Santa Cruz Biotechnology; SCBT, Dallas, TX, USA) for 45 min at 4 °C, immunoprecipitated protein complexes were washed and Laemmli’s sample buffer was added. The samples were boiled and subjected to Western blot analysis. Results shown are representative of three independent experiments.

### 2.3. Immunofluorescence and Proximity Ligation Assay

Transfected or untransfected HeLa cells grown on coverslips were fixed in methanol at −20 °C for 7 min. Samples were incubated with PBS1X containing 4% BSA to saturate non-specific binding and then incubated with primary and secondary antibodies. The nuclei were then counterstained with 4,6-diamino-2-phenylindole (DAPI). Slides were mounted with an anti-fade reagent in glycerol and observed under fluorescent microscopy. The Proximity Ligation Assay (PLA) experiments were performed using the Duolink^®^ in situ Detection Reagents Orange (DUO92007) kit from Sigma-Aldrich. Briefly, saturated (4%-BSA) methanol-fixed cells were incubated with anti-BAF and anti-emerin primary antibodies overnight at 4 °C. Thereafter, slides were incubated for 1 h at 37 °C with secondary probes. Ligation solution was added to each sample and slides were incubated in a humidity chamber for 30 min at 37 °C. Ligation solution was removed with washing buffer and amplification solution was added. Slides were incubated in a humidity chamber for 100 min at 37 °C and then washed with wash buffers. DNA was counterstained with DAPI. Samples were observed by a Nikon Eclipse Ni fluorescence microscope equipped with a digital CCD camera and NIS Elements AR 4.3 software. Quantitative analysis was performed using Duolink Image Tool software (Sigma) by counting 200 nuclei per sample [28]. All images were taken at similar exposures within an experiment for each antibody. Images were processed using Adobe Photoshop (Adobe Systems, San Jose, CA, USA). Results shown are representative of three independent experiments.

### 2.4. Antibodies

The antibodies employed for Western blot analysis or immunofluorescence labeling were: anti-FLAG, mouse monoclonal (Sigma M2 1:1000, 1 h, for Western blot analysis); anti-BAF, rabbit polyclonal (SCBT FL-89, diluted 1:10, overnight at 4 °C for immunofluorescence analysis) anti-BAF, mouse monoclonal (SCBT diluted 1:100, overnight at 4 °C, for the Western blot analysis); anti-prelamin A, goat polyclonal (SCBTSC-6214, used 1:500 for 1 h, for Western blot analysis); anti-lamin A/C, goat polyclonal (SCBTN-18, used 1:100 for 1 h, for Western blot analysis); anti-actin, goat polyclonal (SCBT I-19, diluted 1:1000 for 1 h, for Western blot analysis); anti-emerin mouse monoclonal (Monosan 1084, diluted 1:100 overnight at 4 °C for immunofluorescence analysis and 1:500 for 1 h, for Western blot analysis); anti-P21 rabbit monoclonal (Invitrogen MA5-14949 diluted 1:2000 overnight at 4 °C for Western blot analysis); anti-gamma-H2AX (diluted 1:2000 overnight at 4 °C for Western blot analysis and 1:300 for the immunofluorescence analysis); monoclonal anti-poly Histidine antibody (Invitrogen 4A12E4 diluted 1:00 for 1 h, for immunofluorescence analysis and 1:2000 for 1 h, for Western blot analysis); anti-ERK mouse monoclonal (SCBT sc-94 diluted 1:500for 1h, for the Western blot analysis); anti-p-ERK mouse monoclonal (SCBT sc-7383 diluted 1:500 for 1 h, for Western blot analysis); and anti-β-tubulin mouse monoclonal (Sigma clone TUB 2.1, T4026 diluted 1:1000 for 1 h, for Western blot analysis).

## 3. Results

### 3.1. ROS Generating Agents Affect Emerin Molecular Weight

It is well known that defects in lamin A/C expression affect cellular metabolism. In particular, an increase in free radical (ROS) production is a common feature in prelamin A accumulating cells as well as in lamin A/C-null cells. Starting from this evidence, we wondered if the molecular weight of emerin could be influenced by modifying the cellular amount of ROS. To obtain a ROS-generating environment, HeLa cells were treated with H_2_O_2_, menadione (also known as vitamin K3) or subjected to UV irradiation [32,34,35] (Figure 1a–c). The molecular weight of emerin was then evaluated by Western blot analysis of total lysates from untreated or treated cells during a time-course experiment (Figure 1a–c). H_2_O_2_ and menadione modified the emerin molecular weight with a similar trend (Figure 1a,b, arrowheads). In general, 20 min of chemical stress was sufficient to induce a mobility shift in emerin, whose staining increased during the early stage of the oxidative stress response and disappeared 3.5 h after removal of the chemical stress (Figure 1a,b). Interestingly, a similar result was obtained in HeLa cells subjected to UV irradiation. Under UV light an increased emerin doublet increased up until one hour after treatment (Figure 1c arrowhead).

To assess whether cells were effectively damaged by our treatments (H_2_O_2_, menadione and UV), protein levels of p21 and gamma-H2AX, markers of genotoxic stress were evaluated. It is well known that oxidative stress, affecting DNA at the replication fork, triggers the activation of the translesion DNA repair system (TLS) which impedes the collapse of replication fork, bypassing DNA lesions [36]. A decrease in the amount of p21, concomitantly with an increase in gamma-H2AX detection, demonstrates DNA damage. In HeLa cells subjected to any of the stated treatments, the p21 and gamma-H2AX protein expression patterns were typical of the oxidative stress response (Figure 1a–c). In particular, we observed a rapid P21 decrease during the early phase of the oxidative stress response, while gamma-H2AX became detectable at the end of the initial stage and achieved the maximum amount during the late phase of the stress response (4 h after stress) (Figure 1a–c) [31]. Both p21 and gamma-H2AX returned to baseline by the end of the recovery period (24 h after stress).

The level of Lamin A/C protein is affected during oxidative stress injury. In particular, we observed that lamin A/C staining slightly decreased during the initial stage of the stress response but rapidly returned to normal levels during the recovery phase (Figure 1a–c and Figure S1a). In addition, we observed that the replacement of the cell culture medium, necessary for triggering the stress recovery pathway, could sometimes affect both the amount of lamin A/C and the molecular weight (Figure 1a and Figure S1a), without modifying the molecular weight of emerin or the levels of p21 and gamma-H2AX protein.

Finally, we wondered if simple DNA damage could be responsible for the results described above (Figure S1b). Thus, the molecular weight of emerin was evaluated in HeLa cells treated with doxorubicin, a DNA-intercalating agent, leading to double-strand DNA lesions. Interestingly, we did not observe any change in the amount of emerin over the course of the experiment when analyzed in Western blot, although DNA damage occurring under doxorubicin treatment was confirmed by variations in the levels of gamma-H2AX and P21 protein (Figure S1b).

### 3.2. Emerin Phosphorylation Increase Molecular Weight during the Early Phase of the Oxidative Stress Response

It has been reported that post-translational modifications (PTMs) to emerin (phosphorylation and/or *O*-GlcNAcylation) affect emerin migration in SDS-PAGE gels [24,37,38]. Considering that a slower migrating emerin upper band was obvious in our experimental model, we studied the possible involvement of phosphorylation and *O*-GlcNAcylation in the slower electrophoretic mobility of the emerin upper band during the oxidative stress response. To evaluate the presence of these secondary modifications in our experimental models, HeLa cells were treated with H_2_O_2_ in combination with chemical treatments able to alter protein phosphorylation or inhibit *O*-GlcNAcylation (Figure 2a–d).

HeLa cells co-treated with H_2_O_2_ and a non-specific protein kinase inhibitor (staurosporine) gave the first suggestion that emerin was phosphorylated during the oxidative stress response (Figure 2a, red arrow). In particular, we observed that combining H_2_O_2_ with staurosporine completely abrogated the increase in the molecular weight of emerin even if ROS-induced DNA damage was occurring, as confirmed by a similar decreased expression of p21 protein in both H_2_O_2_- and H_2_O_2_+ staurosporine-treated cells (Figure 2a). The staurosporine protein phosphorylation inhibitory effect was confirmed by a decrease in gamma-H2AX staining in the co-treated cells (Figure 2a).

To further investigate the increase in emerin phosphorylation increase during the oxidative stress response, HeLa cells were treated with H_2_O_2_ in combination with Okadaic Acid (O.A.), a specific inhibitor of serine/threonine protein phosphatases (Figure 2b). In accordance with the previously described results when HeLa cells were treated with O.A. alone, a low level of emerin upper band was observed [39] (Figure 2b, red arrow). On the contrary, combining O.A. with H_2_O_2_ strongly enhanced the level of the upper emerin band (Figure 2b, red arrow), confirming that, under oxidative stress conditions, emerin phosphorylation occurs. In both H_2_O_2_− and H_2_O_2_/O.A.-treated cells, P21 and gamma-H2AX protein levels were in accordance with an ongoing oxidative stress response.

*O*-GlcNAcylation (*O*-GlcNAc) is an additional secondary modification carried-out by the enzyme β-*N*-acetylglucosaminyltransferase (OGT) able to affect the molecular weight of emerin. Since *O*-GlcNAc and phosphorylation of emerin may compete for the same specific protein residues, a fine-tuned crosstalk between these secondary modifications may be necessary [24]. In general, inhibition of *O*-GlcNAc seems to trigger emerin phosphorylation even if a simultaneous increase in both emerin secondary modifications has not been excluded. Starting from the O.A. treatment results, we attempted to understand if emerin GlcNAc could contribute or not to the shift in the molecular weight of emerin during the oxidative stress response (Figure 2c).

In this regard, HeLa cells were treated with a specific inhibitor (OSMI-1) of β-*N*-acetylglucosaminyltransferase alone or in combination with H_2_O_2_ [33]. Western blotting of Emerin showed a modest but detectable increase in the emerin upper band when OSMI-1 alone was added to the HeLa growth medium (Figure 2c, red arrow). On the contrary, the emerin doublet became more prominent when OSMI-1 was combined with H_2_O_2_ treatment (Figure 2c, red arrow). Both p21 and gamma-H2AX protein levels confirmed the ongoing oxidative stress process and the efficacy of OGT [40,41]. These results exclude emerin *O*-GlcNAc under oxidative stress conditions.

To better define if the decrease in *O*-GlcNAc of emerin could improve its phosphorylation during the oxidative stress response, we treated HeLa cells with a combination of H_2_O_2_, O.A., and OSMI-1 (Figure 2d). Using this experimental approach, it was possible to evaluate the molecular weight shift of emerin when both protein *O*-GlcNAc and dephosphorylation were inhibited.

Interestingly, a modest but statistically significant increase in the level of the slower migrating emerin band was detectable when HeLa cells were co-treated with H_2_O_2_, O.A. and OSMI-1 in comparison with the single treatments (Figure 2d, red arrow). The p21 and gamma-H2AX protein amount corroborated with activation of the oxidative stress response, while the increase in immunological staining of phopho-ERK1/2 confirmed the efficacy of O.A. and OSMI-1 in single or combined treatments in the presence of H_2_O_2_ (Figure 2d). Overall, these results strongly suggest that the molecular weight shift in emerin during the initial step of the oxidative stress response is due to protein phosphorylation which is favored by a decrease in *O*-GlcNAcylation (Berk).

### 3.3. Emerin Interaction with BAF Is Affected during the Oxidative Stress Response

BAF is one of the main binding partners of emerin. It has been previously described that emerin–BAF interaction can be modulated by targeting emerin phosphorylation [22,23].

**Figure 3:**
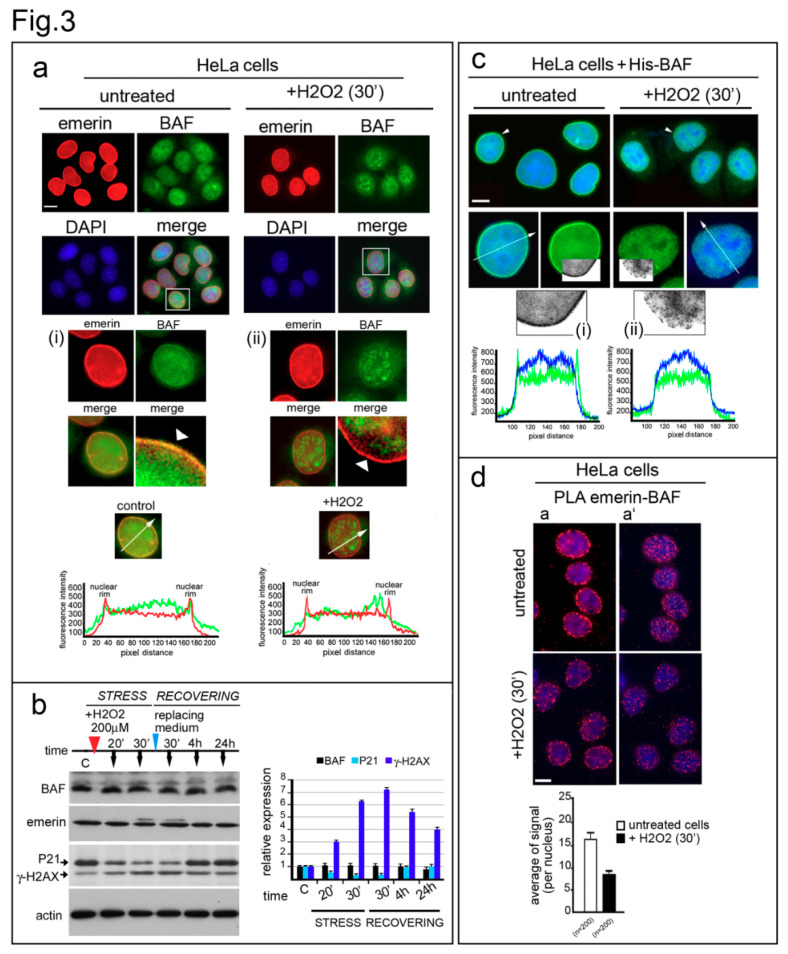
BAF nuclear localization and interaction with emerin is affected during the oxidative stress response. (**a**) Emerin and endogenous-BAF localization in H_2_O_2_-treated HeLa cells. Emerin (red) and endogenous-BAF (green) localization in control (untreated) and treated cells (+H_2_O_2_ (30′)). DNA was counterstained with DAPI (DAPI). Merge of fluorescence signals are shown (merge). In (i) (control) and (ii) (+H_2_O_2_), enlargements of respective nuclei are indicated by squares. BAF nuclear periphery distribution observed in untreated and treated cells is indicated by white arrowheads. Graphs indicate the fluorescence intensity profile along the white arrows. Bar: 20 μm. (**b**) His-tagged BAF localization in control and H_2_O_2_-treated HeLa cells. His-tag immunological staining (green) in control (untreated) and H_2_O_2_-treated cells merged with DAPI is shown. Enlarged images show nuclei (arrowheads). A portion of the nuclear His-BAF staining (green) has been converted to grayscale, and further enlarged, to better demonstrate the difference in His-BAF nuclear distribution observed in control (i) vs. treated cells (ii). The fluorescence intensity profile (white arrows) is reported (graphs). Bar: 20 μm. (**c**) BAF expression during the oxidative stress response. BAF, lamin A/C, emerin, P21, and gamma-H2AX (γ-H2AX) protein levels in untreated HeLa cells (-) or HeLa cells treated (+) with H_2_O_2_ (200 μM) for 30 min and allowed to recover for 24 h. Cells were harvested and lysed at the indicated times (black arrows). Actin was evaluated as a protein loading control. Densitometric analysis of immunolabeled bands is reported. (**d**) Emerin–BAF proximity ligation assay performed in untreated and H_2_O_2_-treated HeLa cells. Complex formation was measured using a rabbit anti-BAF antibody and a mouse anti-emerin antibody. In situ PLA is indicated by the red signal of the rolling cycle amplification products. Nuclei (blue) were counterstained with DAPI. Two different focal planes (a and a’) are shown. The graph indicates the average of positive nuclear spots per nucleus. Scale bars: 20 µm.

BAF immunofluorescence detection was performed in untreated HeLa cells or Hela cells treated with H_2_O_2_. In accordance with previously described results, BAF was equally distributed in both the cytoplasm and the nucleus of untreated cells, even if a different intensity in the nucleoplasm and nuclear lamina staining was observed among different cells (Figure 3a). On the contrary, in H_2_O_2_-treated cells, the nuclear distribution of BAF changed: association with the nuclear lamina was reduced and a prevalent re-localization to intranuclear speckles was observed (Figure 3a, arrowheads). In accordance, BAF immunological staining was completely excluded from gamma-H2AX-positive regions (Figure S2). Finally, no change in either the amount of BAF protein or the localization of emerin was observed during the oxidative stress response (Figure 3a,b).

To confirm our findings, a His-tagged-BAF construct was transiently expressed in HeLa cells, and its nuclear distribution was evaluated under stressing conditions (Figure 3c). In untreated cells, the exogenous protein was detectable both at the nuclear periphery and in the nucleoplasm (Figure 3c) [26]. As observed for the endogenous protein, His-BAF was prevalently recruited in the nucleoplasm upon treatment with oxidative stress stimuli, while protein staining at the nuclear periphery was reduced (Figure 3c, i and ii). However, it is important to note that both endogenous BAF and His-tagged BAF recovered their normal nuclear distribution at the end of the stress-pathway response (Figure S3a,b).

Since BAF intranuclear localization dynamics suggests a decrease in emerin–BAF binding during the initial steps of the oxidative stress response, we performed a Proximity Ligation Assay (PLA) to evaluate in situ the emerin–BAF interaction during ROS injury (Figure 3d and Figure S4). We observed a detectable decrease in emerin–BAF association (Figure 3d and Figure S4). This finding was confirmed by a co-immunoprecipitation assay performed in HeLa cells expressing His-tagged-BAF (Figure S5). Evaluation of His-tagged protein from control and H_2_O_2_-treated cells, using immunoprecipitation and Western blotting, showed a decrease in the emerin–BAF interaction under oxidative stress condition.

### 3.4. EDMD1 Cells Are More Sensitive to ROS-Induced DNA Damage

**Figure 4:**
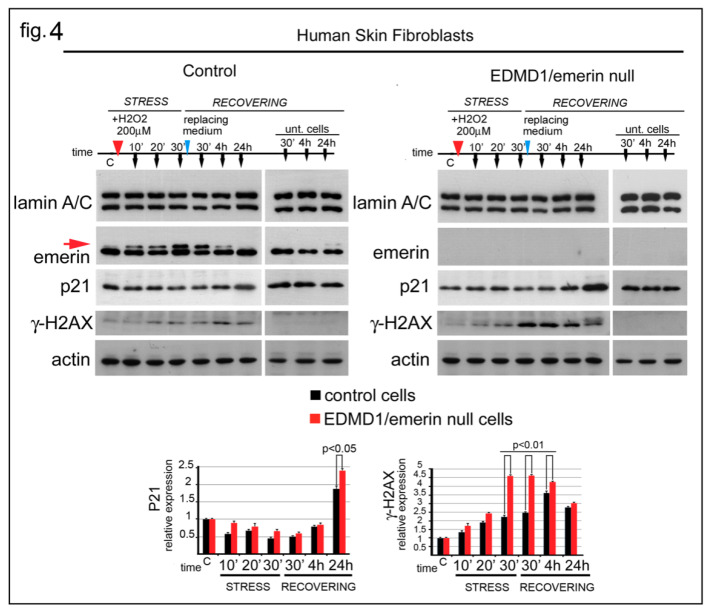
Emerin depletion affects the DNA-damage response. Western blot analysis of human skin fibroblasts from a healthy donor (Control) and an EDMD1 patient (EDMD1/emerin-null) subjected to H_2_O_2_ treatment. Experimental procedure and sample collection are indicated over each Western blot panel. Stress (STRESS) indicates the time (30 min) during which cells underwent stressing stimulus. Red arrowhead indicates H_2_O_2_ addition to the growth medium. “Recovering” indicates the time after replacement of growth medium (blue arrowheads). Sample collection at the different timepoint is indicated by black arrows. Time is indicated in minutes during the stress (10′, 20′, and 30′) and in minutes (30′) and hours (4 and 24 h) during the recovery. “C” indicates untreated cells before treatment. “Unt. Cells” indicates untreated cells subjected to growth medium replacement and harvested after 30 min, 4 h and 24 h of recovering. Lamin A/C, emerin, p21, gamma-H2AX (γ-H2AX), emerin, and actin bands are shown. Densitometric analysis of p21 and gamma-H2AX (γ-H2AX) bands normalized to control and EDMD1 untreated cells is reported. The red arrow indicates the phosphorylated emerin band. Statistical differences (Student’s t-test) between control and treated EDMD1 cells are indicated.

The above reported results strongly suggest that emerin might play an important role during the oxidative stress response. To better explore this issue, P21 and gamma-H2AX were evaluated in emerin-null cells subjected to oxidative stress (Figure 4). Human skin fibroblasts from a healthy donor and an EDMD1 patient were treated with H_2_O_2_ in a time-course experiment (Figure 4). Western blotting analysis showed that in control fibroblasts the upper emerin band increased following the same trend observed in HeLa cells (Figure 4, red arrow). The upper emerin band was detectable early (10 min) after H_2_O_2_ addition and increased over the next 30 min. As observed in HeLa cells, as well as in human fibroblasts, the upper emerin band disappeared at the end of the recovery process (24 h) (Figure 4, red arrow). Interestingly, evaluation of the level of p21 protein during stress showed a different modulation in control cells compared to EDMD1/emerin-null cells (Figure 4). In both untreated cell cultures, the level of p21 was similar, while a detectable initial decrease in p21, with subsequent partial restoration of the level of p21, was observed in control fibroblasts subjected to H_2_O_2_ treatment. On the contrary, in EDMD1 cells, a decrease in the level of p21 was barely detectable at the initial stage of the oxidative stress response while a higher level of p21, relative to control cells, accumulated at the end of the recovering phase (Figure 4). The evaluation of DNA damage markers during the oxidative stress response showed that EDMD1 cells accumulated more gamma-H2AX than control fibroblasts. However, in both control and EDMD1 fibroblasts, gamma-H2AX became barely detectable 24 h after H_2_O_2_ treatment (Figure 4).

### 3.5. Emerin Is Also Affected in Various LaminA Deficiency Conditions

**Figure 5:**
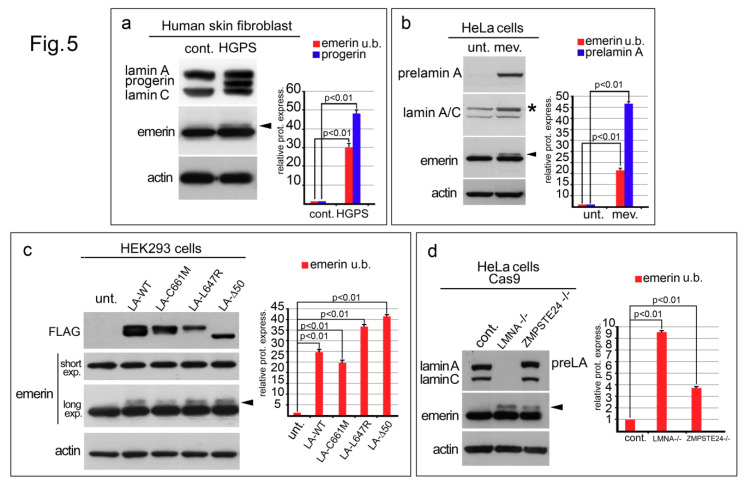
Prelamin A processing defects or lamin A/C silencing affect emerin expression. (**a**) Western blotting of human skin fibroblasts from a healthy donor (cont.) and HGPS patient (HGPS). Lamin A, progerin and lamin C were detected using a goat polyclonal anti-lamin A/C antibody. Emerin staining at predicted molecular weight is observed in control cells while in HGPS cells an additional emerin band is detected (arrowhead). (**b**) Total lysates from untreated HeLa cells (unt.) or HeLa cells treated with mevinolin (mev.) were subjected to prelamin A (prelamin A), lamin A/C, and emerin immunoblotting. Prelamin A band, detected by the anti-lamin A/C antibody is indicated by an asterisk (*). The upper (phosphorylated) emerin band observed in mevinolin treated cells is indicated by an arrowhead. (**c**) Emerin detection in HEK293 cells transiently expressing Flag-tagged prelamin A constructs. Total lysates of untransfected (unt.) HEK293 cells or HEK293 cells expressing wild-type prelamin A (LA-WT), non-farnesylated prelamin A (LA-C661M), farnesylatedand carboxymethylated prelamin A (LA-L647R), or progerin (LA-∆50) were probed with antibodies specific for FLAG (FLAG) and emerin (two different exposure, short and long, are shown). The upper (phosphorylated) emerin band is indicated by an arrowhead. (**d**) Total lysates from untreated HeLa cells (cont.) or HeLa cells subjected to CRISPR/Cas9 genome editing for the *LMNA* (LMNA −/−) or *ZMPSTE24* (ZMPSTE24 −/−) gene deletion were probed with antibodies specific for lamin A/C and emerin. The upper (phosphorylated) emerin band is indicated by an arrowhead. In (**a–d**), actin was evaluated as a protein loading control. The densitometric analysis of immunolabeled bands is shown. Statistical differences (Student’s t-test) between control cells and cells bearing prelamin A processing defects or depleted in lamin A/C, are indicated.

Emerin was evaluated by Western blotting analysis in different cellular models characterized by defects in lamin A/C expression and a previously described “ROS-generating environment” (Figure 5). In human skin fibroblasts from a healthy donor, the emerin protein band was detectable at the predicted molecular weight as a single band (Figure 5a). However, in HGPS cells, an upper emerin band was observed (Figure 5a, arrowhead). A similar result was obtained when HeLa cells were forced to accumulate prelamin A through mevinolin treatment (Figure 5b). A sharp upper emerin band became visible in treated cells (Figure 5b, arrowhead). Both anti-prelamin A and anti-lamin A/C antibody staining confirmed the accumulation of the non-farnesylated lamin A precursor in mevinolin-treated cells (Figure 5b). The molecular weight change in emerin was also observed in HEK293 cells expressing FLAG-tagged prelamin A constructs (Figure 5c). Total lysates from cells expressing wild-type lamin A (LA-WT) or prelamin A mutants, including non-farnesylated prelamin A (LA-C661M), farnesylated and carboxymethylated prelamin A (LA-L647R), or progerin, which is a truncated form of farnesylated and carboxymethylated prelamin A (LA-∆50), showed an upper emerin band in Western blotting (Figure 1c, arrowhead). Finally, a similar emerin profile was detected in HeLa cells subjected to lamin A/C or ZMPSTE24 silencing by the CRISPR-Cas9 genome editing technique (Figure 5d). In lamin A/C-null cells and in prelamin A accumulating cells, the anti-emerin antibody revealed an upper emerin band which was undetectable in control cells (Figure 5d, arrowhead).

## 4. Discussion

Our work shows, for the very first time, that oxidative stress modifies emerin in a rapid and highly reproducible way. The molecular weight of emerin increases during the early phase of the response to free radicals and returns to baseline levels when the DNA damage is repaired. Concomitantly, the emerin–BAF interaction decreases, prevalently favoring BAF nucleoplasmic localization during the initial stage of the stress response.

Oxidative stress is one of the best characterized detrimental effects due to perturbation of the nuclear lamina [42]. Starting from results obtained in FPLD2 human cells, showing how the accumulation of a mutated form of prelamin A triggers mitochondrial dysfunction [3], evidence has demonstrated that defects in the nuclear lamina influence both ROS production and antioxidant defense [42]. It is well known that accumulation of both prelamin A-forms and mature A-type lamin depletion cause a free radical generating environment with a prominent increase of ROS in lamin A/C silenced cells. Interestingly, a link between nuclear lamina defects and alterations in the molecular weight of emerin has been previously demonstrated. In particular, a study performed in human fibroblasts harboring an *LMNA* mutation, which led to the absence of A-type lamins, showed, by Western blotting analysis, a clearly detectable emerin doublet [16]. Here, we show that oxidative stress affects the molecular weight of emerin in particular; an upper emerin band representing phosphorylated emerin appears early during the oxidative stress response and rapidly disappears during recovery. Interestingly, BAF changes its intranuclear distribution following the same timing, suggesting that a dynamic modification of both emerin secondary modifications and BAF nuclear distribution could be a part of the same nuclear lamina-located stress-sensing pathway.

Our results suggest that a finely tuned balance between emerin phosphorylation and *O*-GlcNAcylation occurs under oxidative stress condition. Our in vivo results, obtained by selective inhibition of dephosphorylation and/or *O*-GlcNAcylation, show that emerin phosphorylation is enhanced when intracellular ROS levels increase. Indeed, we observed that combining H_2_O_2_ with staurosporine prevents change in the molecular weight of emerin while blocking protein dephosphorylation or *O*-GlcNAcylation makes the upper emerin band more evident. These results can be explained by taking into account the model proposed by Berk and co-workers [24]. According to these authors, *O*-GlcNAcylation and phosphorylation may compete for the same emerin regions affecting emerin conformation and/or protein-protein interaction in an alternative way. Interestingly, emerin phosphorylation versus *O*-GlcNAcylation (on Ser-173) has an opposite effect on emerin–BAF interaction: in general, when emerin phosphorylation increases, the release of BAF is favored [24]. Thus, the BAF nucleoplasmic localization we describe during the early phase of the oxidative stress response can be considered an indirect demonstration of emerin phosphorylation being responsible for the doublet observed.

Change in BAF intranuclear localization in response to ROS increase is an intriguing finding considering that this DNA binding protein is an epigenetic regulator and a chromatin organizer [43,44]. Our observation about “BAF shuttling” from the nuclear envelope to genome suggests a possible mechanism in which the emerin–BAF protein complex could be necessary not only to “sense” environmental stress but, more importantly, also to promote cell survival by modifying gene expression. By targeting the phosphorylation of emerin, ROS could trigger the release of BAF from the nuclear envelope to specific chromatin domains where, by influencing secondary modifications of histones, it could promote the expression of cell survival and antioxidant genes. In accordance, it has been previously described that BAF phosphorylation and nuclear localization change in *C.elegans* larvae subjected to stress stimuli such as caloric restriction and heat shock, supporting a scenario in which, by targeting the emerin–BAF interplay, it could be possible to modulate a specific response to stress [45]. In this regard, it should be mentioned that emerin becomes tyrosine phosphorylated in response to mechanical force and mediates a series of cellular modifications necessary for a proper response to the mechanical stress, which are absent in emerin mutated cells [46,47]. However, what kind of molecular role BAF plays in such cellular mechanism is still unknown.

Among cellular mechanisms counteracting the detrimental effects of ROS, the DNA damage response is one of the most important. Increased ROS triggers the activation of cell repair systems, responsible for fixing both DNA-single-strand breaks (SSB) and double-strand breaks (DSB), and, interestingly, some of them have been related to the emerin–BAF protein complex [35,44]. In our experimental model, we observed that the emerin–BAF complex was “remodeled” early after H_2_O_2_, menadione, and UV treatments of the cells. In particular, we observed that the emerin-phosphorylated band appeared before gamma-H2AX detection and concomitantly with a decrease in p21. However, not all DNA damaging agents trigger emerin phosphorylation, as demonstrated by our results obtained using doxorubicin, in which the emerin doublet was undetectable, even if DNA damage was present. This is an important finding considering that H_2_O_2_, menadione and UV irradiation are all known to cause DNA-SSB-lesions (typically repaired by base excision repair (BER) or nucleotide excision repair (NER) systems) [48], while doxorubicin induces DSB-lesions [49], suggesting a preferential involvement of the inner nuclear membrane complexes in SSB-DNA repair pathways [50]. In accordance, a dynamic BAF–emerin interaction with molecular elements of the NER system has been previously demonstrated. This cellular “machinery” recognizes and repairs helical distortions in the DNA duplex and modifications of the DNA chemistry [44]. The NER process requires the action of more than 30 proteins in a stepwise manner that includes damage recognition, local opening of the DNA duplex around the lesion, dual incision of the damaged DNA strand, gap repair synthesis, and strand ligation. Briefly, following UV exposure, the DDB1–DDB2–CUL4A–RBX1 complex (DDBCUL4) localizes to the site of damage and ubiquitinates XPC and DDB2 [51,52]. Polyubiquitination of DDB2 reduces its affinity for damage while XPC remains unaffected promoting lesion repair. Interestingly, both emerin and BAF show an in vivo dynamic interaction with DDB2 and CUL4A, which is differentially regulated by UV-damaged DNA. In particular, interaction of emerin and BAF with DDB2 is observed in both control and UV-treated cells while the binding with CUL4 is detectable exclusively in untreated cells [44], suggesting a possible inner nuclear membrane role in the storage/surveillance of NER proteins. In accordance, the involvement of the inner nuclear membrane protein LEM2 in the function of NER has been recently described [50].

Even if experimental data about the emerin–BAF protein complex involvement in both DNA-BER and DNA-TLS are missing, our results obtained in H_2_O_2_-treated EDMD1 cells strongly suggest that these DNA-damage repair mechanisms could also be influenced by the inner nuclear membrane proteins. Emerin-null cells subjected to ROS injury were able to recover from DNA damage but with an evident delay in the process-progression. In particular, the amount of p21 is higher both during the early and late phases of the damage response, and the gamma-H2AX amount was expressed at a higher level and detected for a longer period of time in EDMD1-cells than in control H_2_O_2_-treated fibroblasts. This could be due to a less efficient degradation of p21 protein or, alternatively, more prominent DNA damage affecting EDMD1 cells during the early stage of the stress response. In this regard, a direct role for BAF in the regulation of PARP1 activity in the oxidative stress-related DNA damage response has been recently demonstrated [53].

Finally, after evaluating emerin expression in cellular models with lamin A expression defects, we observed the same emerin–SDS migration pattern observed in cells subjected to oxidative stress. In particular, in skin fibroblasts from an HGPS patient as well as in fibroblasts from a healthy donor forced to accumulate prelamin A (by mevinolin treatment), or, in HEK293 cells expressing different prelamin A forms, including progerin, an additional upper emerin band was detectable. Again, replicating a lamin A-null condition or the exclusive expression of prelamin A, in the absence of mature lamin-A, by the CRISPR-Cas9 technique, the emerin molecular weight was similarly altered. Even if additional experiments must be performed to better define the phosphorylation status of emerin in the models described above, our observations strongly suggest an increase in emerin phosphorylation resulting from the “ROS-generating environment” of laminopathic cells.

However, it is important to note that increased emerin phosphorylation has also been observed during muscle differentiation, a normal physiologic event [54]. Interestingly, during myogenesis intensive metabolic remodeling occurs, mitochondrial content and respiratory chain activity increases and ROS production is favored [55]. Thus, the modification of the emerin–BAF protein complex could be necessary not only for modifying gene expression to promote cell survival during the oxidative stress response, but also to mediate the so-called “ROS-regulatory function” in normal cells.

Overall, our findings demonstrate that the emerin–BAF protein complex is modified during the early phase of the oxidative stress. We show, for the very first time, a rapid and transient modification of the emerin–BAF complexes at a well-defined moment of stress injury. The rapid and temporary change of emerin and BAF behavior under stressing conditions is the most interesting finding of our work, which points out the importance of the inner nuclear membrane as a sensor/mediator of external stimuli that must be rapidly transduced in the nucleus or “fixed” as in the case of the DNA-damage.

## Supplementary Materials

The following can be found at https://www.mdpi.com/2073-4409/9/6/1415/s1. Figure S1. (a) Densitometric analysis of lamin A/C bands shown in the Western blotting analysis in Figure 1a–c. (b) Western blot analysis of doxorubicin-treated cells. Cells treated with 2 μM doxorubicin (Doxo) were harvested and analyzed during a time-course experiment at the indicated time (10 min, 20 min, 30 min, 1 h and 4 h). Untreated cells (-) were processed after 30 min and 4 h. Lamin A/C, emerin, P21, gamma-H2AX (γ-H2AX) and actin bands are shown. Densitometric analysis of P21 and gamma-H2AX is shown. Figure S2. (a) Immunofluorescence evaluation of BAF (green) and gamma-H2AX (γ-H2AX, red), in untreated HeLa or H_2_O_2_-treated HeLa cells. In merge, BAF and gamma-H2AX double immunolabel staining is shown. (b) Enlargement of BAF/gamma-H2AX double staining observed in the nucleus is indicated by an arrowhead in A. Graphs indicate the fluorescence intensity profile along the white arrow. Bar 20 μm. Figure S3. (a) Emerin and BAF localization in HeLa cells subjected to H_2_O_2_ treatment. Merged image of emerin (red) and BAF (green) immunofluorescence staining detected in untreated (untreated), H_2_O_2_-treated and stress-recovered cells (recovery (4 h)). Enlarged images show BAF localization (green) in cells indicated by squares. (b) His-tagged BAF localization in Hela cells subjected to H_2_O_2_ treatment. Merge image of His (green) and DAPI (Blue) staining detected in untreated (untreated), H_2_O_2_-treated cells and stress-recovered cells (recovery (4 h)). Enlarged images show His-BAF localization (green) in cells indicated by arrowheads. Figure S4. Evaluation of emerin–BAF interaction by Proximity Ligation Assay. In situ PLA performed in untreated HeLa cells (untreated) or H_2_O_2_-treated cells is indicated by the red signal. Nuclei (blue) were counterstained with DAPI. Two different focal planes (a and a’) are shown. Squares indicate the fields shown enlarged in Figure 3b. Figure S5. Western blot analysis of a co-immunoprecipitation experiment performed in HeLa cells transiently transfected with a His-tagged-BAF construct (HeLa cells + His-BAF). His-BAF was immunoprecipitated in untreated (−) or H_2_O_2_-treated (+) cells, and the endogenous emerin interaction was detected. Western blotting analysis of emerin and His-BAF proteins in whole lysate (input) and co-immunoprecipitated complexes (IP-His) are shown. No protein staining was observed in absence of anti-His antibody (A/G protein). Emerin upper band observed in treated cells is indicated by red arrowhead. Quantitative analysis of co-immunoprecipitated emerin, normalized to immunoprecipitated His-BAF, is shown.
