# Supplementary figures and images for: Emerin Phosphorylation during the Early Phase of the Oxidative Stress Response Influences Emerin–BAF Interaction and BAF Nuclear Localization
Source: Cells. 2020 Jun 6;9(6):1415. doi: 10.3390/cells9061415 (PMC7349582; doi:10.3390/cells9061415)

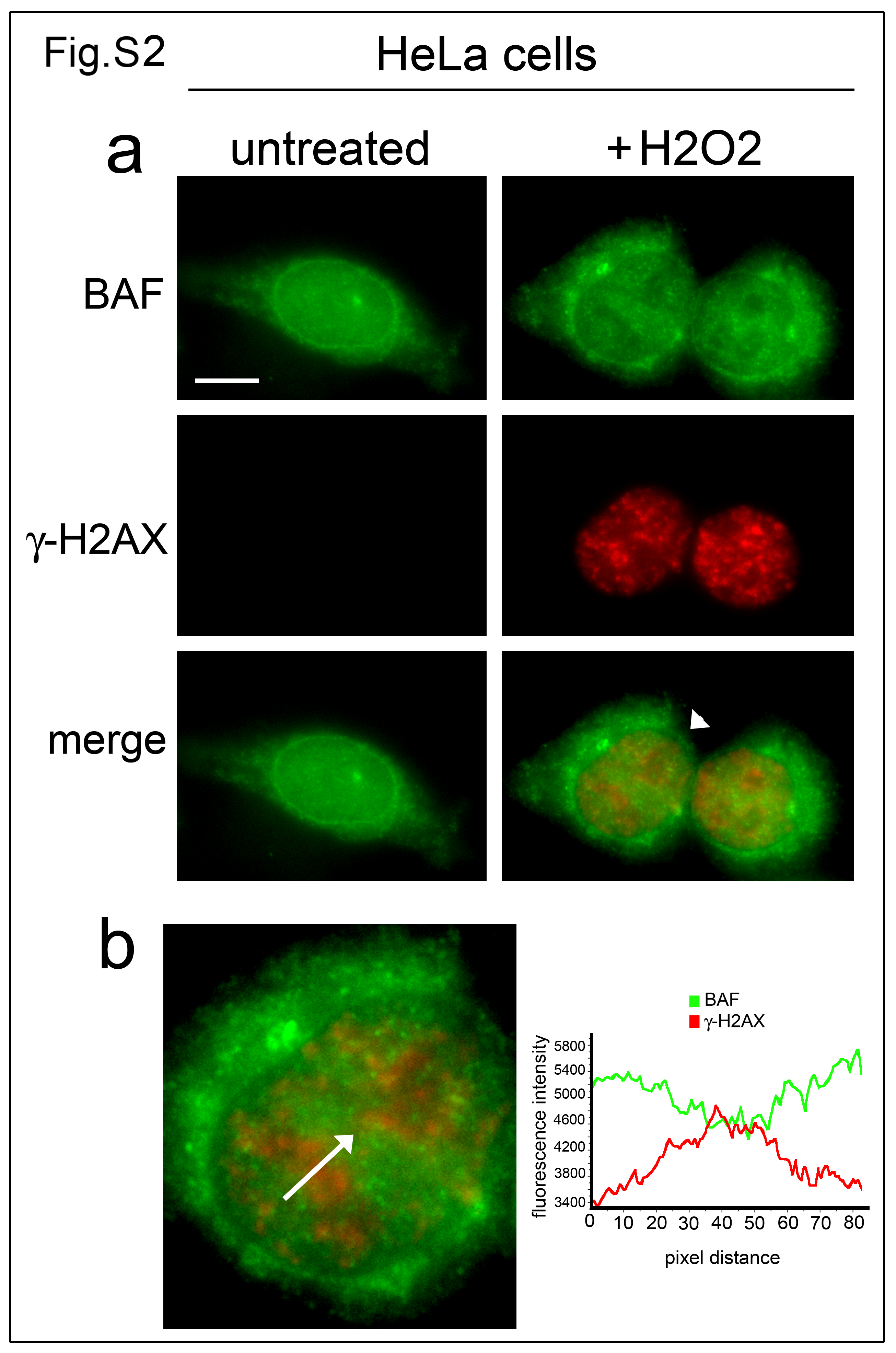

Supplement: Supplementary file 1 [file cells-09-01415-s001.zip › Cenni figure S2 revised.tif]

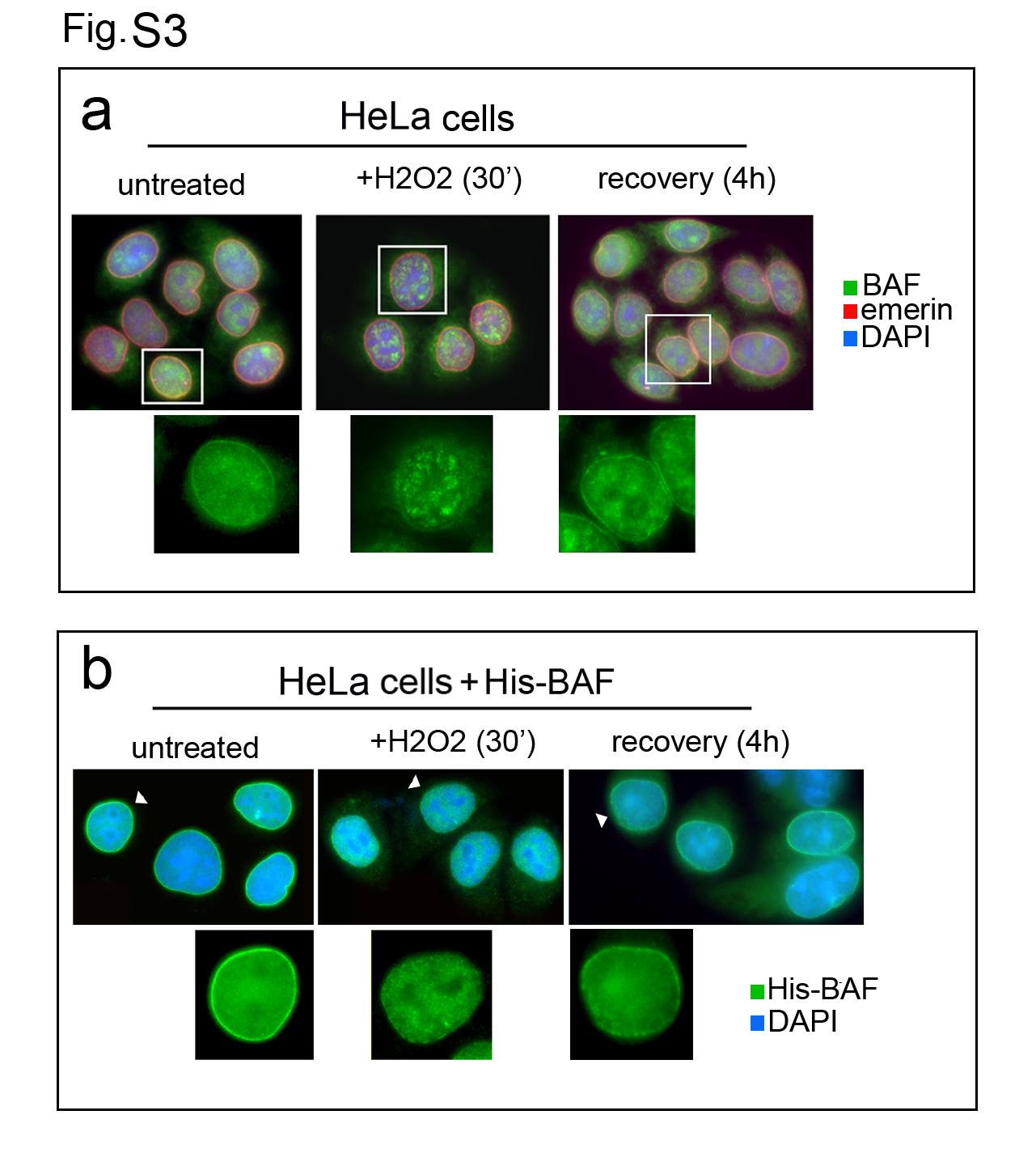

Supplement: Supplementary file 1 [file cells-09-01415-s001.zip › Cenni Figure S3 revised.tif]

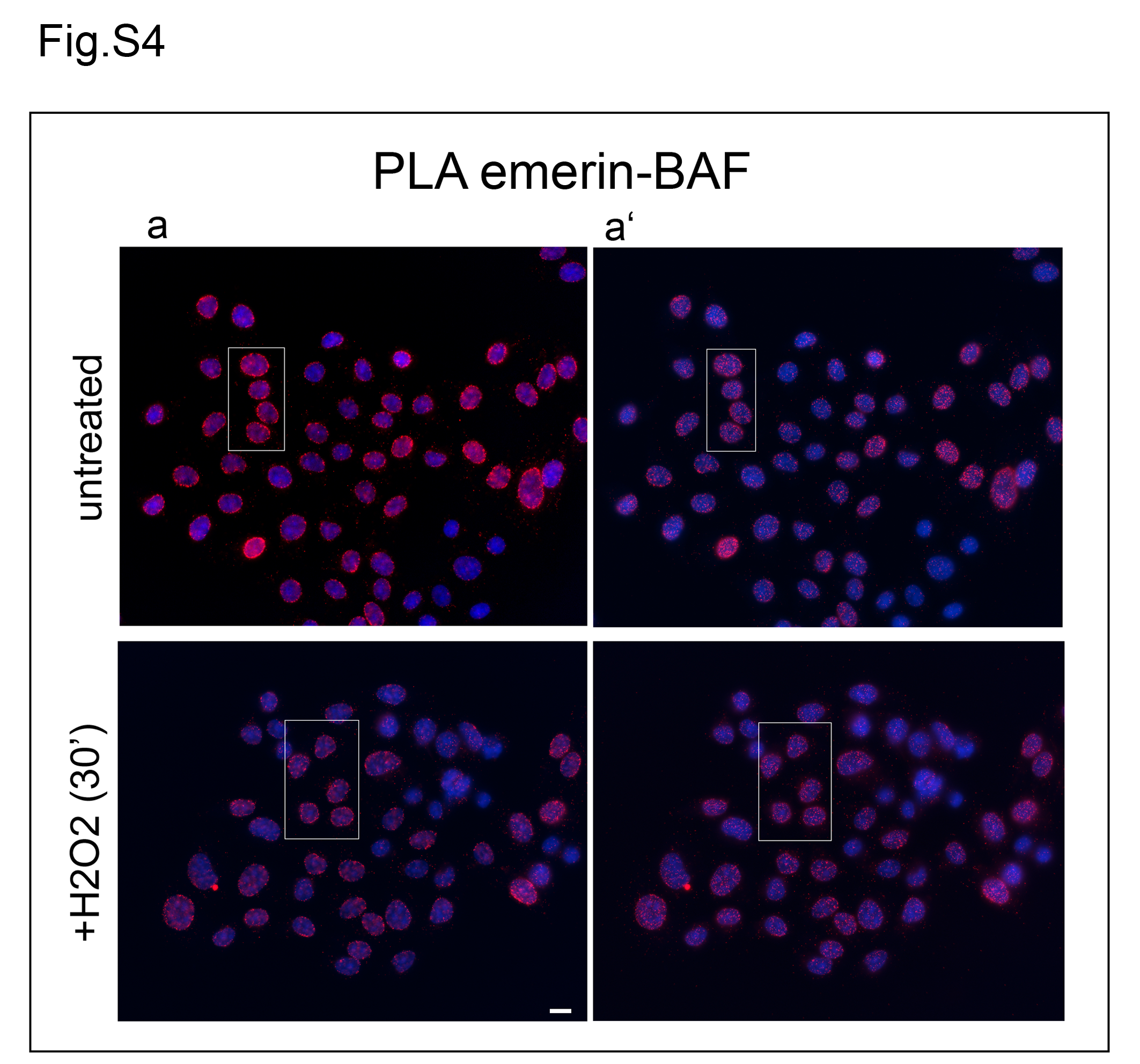

Supplement: Supplementary file 1 [file cells-09-01415-s001.zip › Cenni Figure S4 revised.tif]

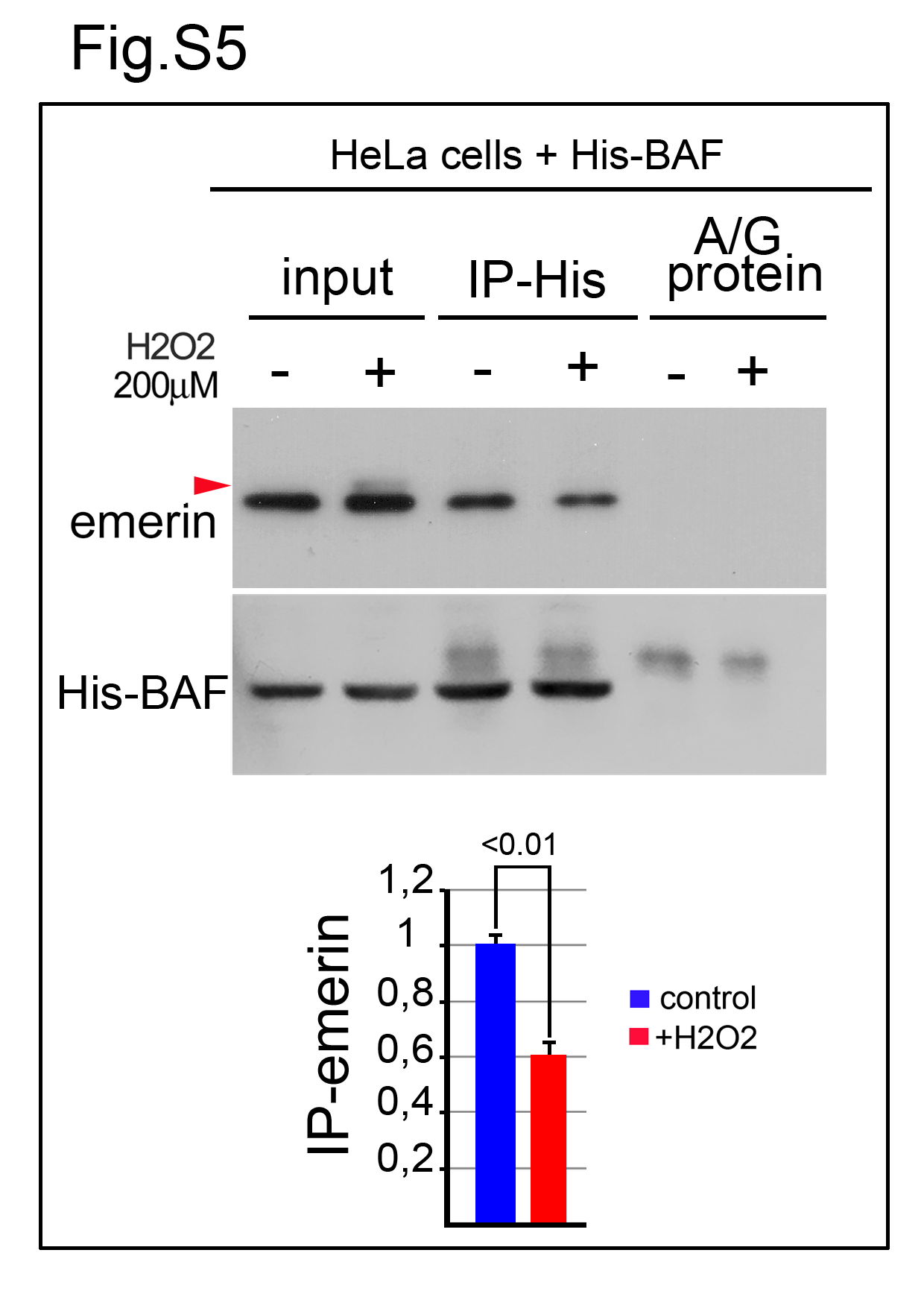

Supplement: Supplementary file 1 [file cells-09-01415-s001.zip › Cenni figure S5 revised.tif]

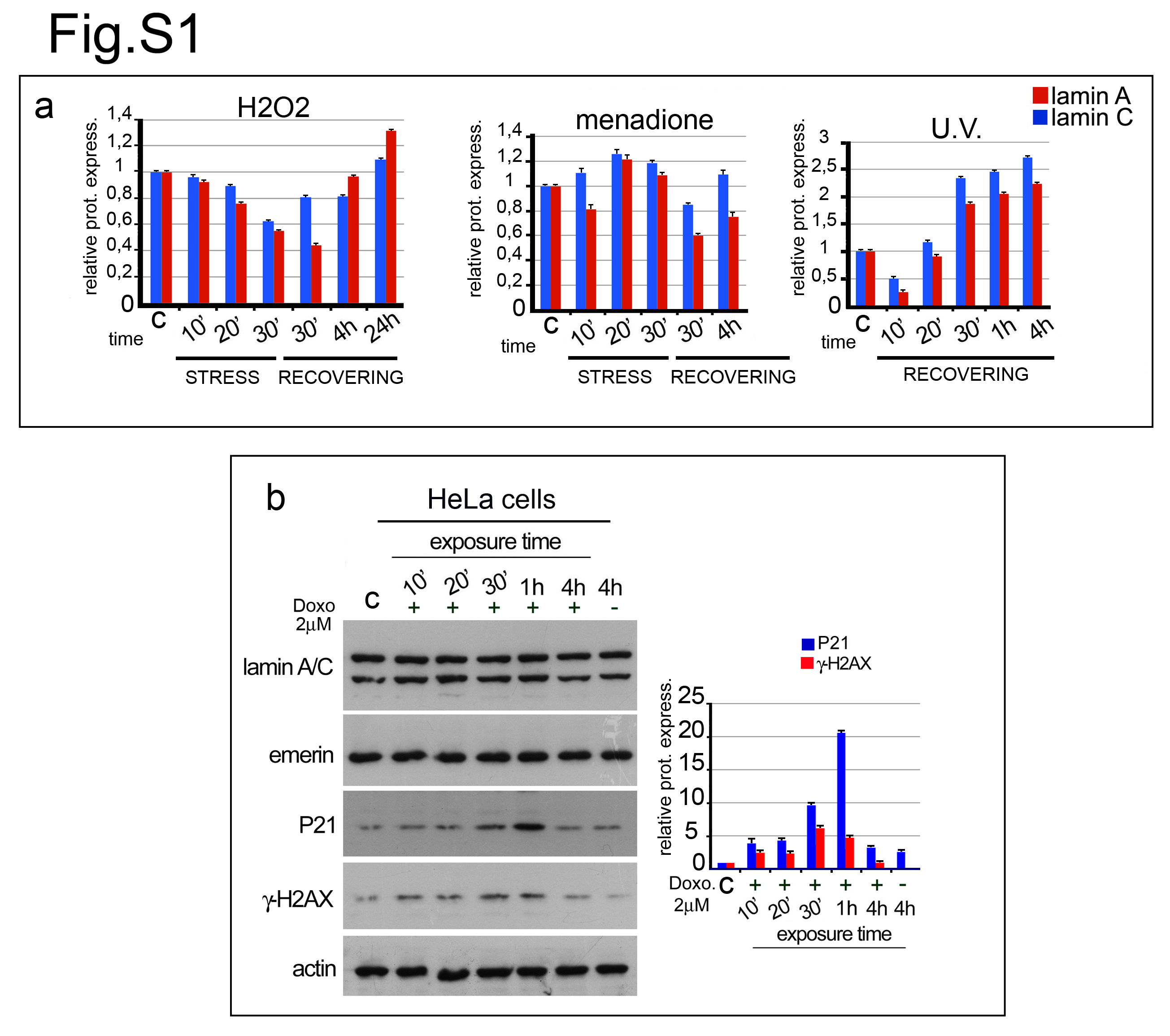

Supplement: Supplementary file 1 [file cells-09-01415-s001.zip › Cenni figure S1 revised.tif]
